# Melittin inactivates YAP/HIF-1α pathway via up-regulation of LATS2 to inhibit hypoxia-induced proliferation, glycolysis and angiogenesis in NSCLC

**DOI:** 10.1016/j.clinsp.2024.100407

**Published:** 2024-06-17

**Authors:** Hao Li

**Affiliations:** Department of Blood Transfusion, Shandong Provincial Hospital affiliated to Shandong First Medical University, Jinan, Shandong, China

**Keywords:** Melittin, LATS2, Proliferation, Glycolysis, Angiogenesis, Non-small cell lung cancer

## Abstract

**Background:**

NSCLC is one of the most common causes of death. The hypoxia microenvironment contributes to cancer progression. The purpose was to explore the effects and mechanism of melittin on NSCLC cells in the hypoxic microenvironment.

**Methods:**

NSCLC cell lines (A549 and H1299) were cultured in normoxia or hypoxia conditions with or without melittin treatment. The viability of the cells was detected via MTT assay and the proliferation ability was evaluated by EdU assay. QRT-PCR was performed to evaluate GLUT1, LDHA, HK2, VEGF and LATS2 mRNA levels. Glucose transport was assessed by the 2-NBDG uptake assay. The angiogenesis was determined by the tubule formation assay. The protein expressions of GLUT1, LDHA, HK2, VEGF, LATS2, YAP, p-YAP and HIF-1α were detected via western blotting assay. The tumor formation assay was conducted to examine the roles of melittin and LATS2 *in vivo*.

**Results:**

Melittin inhibited hypoxia-induced cell viability, proliferation, glycolysis and angiogenesis as well as suppressed YAP binding to HIF-1α in NSCLC. Melittin inactivated the YAP/HIF-1α pathway via up-regulation of LATS2, ultimately inhibiting cancer progression of NSCLC. Moreover, melittin suppressed tumor growth via up-regulation of LATS2 *in vivo*.

**Conclusion:**

Melittin inactivated the YAP/HIF-1α pathway via up-regulation of LATS2 to contribute to the development of NSCLC. Therefore, melittin is expected to become a potential prognostic drug for the therapy of NSCLC.

## Highlights

• Melittin inhibits hypoxia-induced proliferation, glycolysis and angiogenesis in non-small cell lung cancer.
• The activation of YAP/HIF-1α pathway promotes non-small cell lung cancer.
• Melittin inactivates the YAP/HIF-1α pathway by up-regulating LATS2 expression.

## Introduction

Lung cancer is common in both men and women, and it has the highest mortality rate all over the world.^1^ According to the metastatic ability, lung cancer is divided into small cell lung carcinoma (SCLC) and non-small cell lung carcinoma (NSCLC). Among all lung cancer patients, most are NSCLC (85 %).^2^ Therefore, finding out more effective drugs and determining the mechanism are important goals. The development of the tumor includes ten characteristics, abnormal energy supply and continuous angiogenesis are two of them^3^ which play important roles in tumor progression, including tumor growth, invasion and metastasis.^4^ Hypoxia is commonly detected in the tumor microenvironment. By anaerobic glycolysis, the tumor metabolizes glucose to lactic acid and survives in an anoxic environment. Hypoxia leads to reduced oxidation of mitochondrial substrates and increased glycolysis.^5^ This has also been demonstrated in NSCLC.^6^ What's more, hypoxia is also a major stimulator of angiogenesis.^7^^,^^8^ Hypoxia-Inducible Factor (HIF)-1α, controls homeostatic response to hypoxia via activation of gene transcription.^9^ Consequently, cells lacking HIF-1α fail to upregulate glycolytic enzymes and lactic acid production under hypoxia.^10^ HIF-1α targeted genes, Glucose Transporter (GLUT1), Hexokinase II (HK2), Lactate Dehydrogenase A (LDHA), are critical for increased glucose uptake and catabolism and are strongly implicated in tumorigenesis.^11^, ^12^, ^13^ Furthermore, the HIF-1α targets the promoter of the Vascular Endothelial Growth Factor (VEGF) to trigger its expression and the subsequent angiogenesis.^6^

Melittin is extracted from honeybee venom.^14^ It is a medicine that has been widely used in the treatment of a variety of diseases, such as anti-tumor,^14^ anti-inflammation^15^ and anti-microbial property.^16^ Researchers have found that melittin induces NSCLC apoptosis and decreases invasion and migration abilities via inhibition of miR-183.^17^ However, the effects of melittin on glycolysis and angiogenesis in NSCLC have not been fully elucidated.

Here we set up the *in-vitro* hypoxia NSCLC cell model to explore the effects of melittin on glycolysis and angiogenesis and investigate the molecular mechanism of melittin NSCLC.

## Materials and methods

### Cell culture

The NSCLC cell lines (A549 and H1299) and Human Endothelial Cells (HUVEC) were obtained from ATCC (USA). These cells were cultured in DMEM/F12 medium (Gibco, USA) supplemented with 10 % FBS, 100 U/mL p/s. All cells were incubated with 5 % CO_2_. For the hypoxia treatment, the cells were then attached to the dishes in normoxia condition for 12h. The dishes were sent to hypoxia conditions (1 % O_2_, 5 % CO_2_, and 94 % N_2_; Lishen, China). After being incubated for 24h, the cells were collected.

### Cell transfection

The si-LATS2 and the Negative Controls (si-NC) were assembled from Genepharma (Shanghai, China). When the confluence reached 80 %, the Lipofectamine 3000 (Invitrogen, USA) was chosen to perform transfections. After 24h transfection, cells were harvested.

### MTT assay

Cell viability is defined as a vital indicator to detect the proliferation of cells. The viability of A549 and H1299 cells was detected by MTT assay. Cells were seeded onto 96 well-plated and allowed to attach. Media were then discarded and replaced with a new medium containing 2 μg/mL melittin and cultured for 24, 48 and 72 h. MTT was added to each well for 4 h incubation. At the end of the experiment, 100 μL DMSO was added, and the absorption was measured in the microplate at 490 nm.

### EdU assay

The EdU assay was used to measure cell proliferation. The A549 and H1299 cells were plated into 24-well plates. After being treated with melittin for 48h, cells were washed with PBS. The proliferation of NSCLC cells was quantified *in vitro* by EdU DNA Proliferation and Detection kit (RiboBio, China) and nucleis were stained with DAPI (Beyotime, China) following the manufacturer's protocol. Images were taken with a fluorescence microscope (Olympus, Japan).

### QRT-PCR

Total RNAs were isolated from A549 and H1299 cells using a Total RNA Extractor Kit (Sangon, China). Then RNA was reverse transcribed into cDNA using the cDNA Synthesis Kit (Beyotime, China). Next, qRT-PCR was conducted using SYBR qPCR Master Mix (Beyotime, China) on a CFX96 q-PCR system (Bio-Rad, USA). The relative expression was calculated using the 2^−ΔΔCt^ method. The following primers were used for qRT-PCR: HK2, forward: 5’-TACAGTCAACGTCATCTGAACTG-3’ and reverse: 5’-CGTACTTGTCGTATTCAAGATGC-3’; LDHA, forward: 5’-ATGAAGGACTTGGCGGATGA-3’ and reverse: 5’-ATCTCGCCCTTGAGTTTGTCTT-3’; GLUT1, forward: 5’-ATGGATCCAGCAGCAAGAAGGCGC-3’ and reverse: 5’-GATGCCAACGACGATTCCCAGCT-3’; VEGF, forward: 5’-TGCTGTCTTGGGTGCATTGG-3’ and reverse: 5’-AGGTCTCGATTGGATGGCAG-3’; LATS2, forward: 5’-CAGGATGCGACCAGGAGATG-3’ and reverse: 5’-CCGCACAATCTGCTCATTC-3’; GAPDH, forward: 5’-GGGCTCTCTGCTCCTCCCTGT-3’ and reverse: 5’-ACGGCCAAATCCGTTCACACC-3. The relative expressions were normalized by GAPDH.

### Western blot

The protein was extracted, and the concentrations were determined by the BCA Protein Assay Kit (Thermo Fisher, USA). The samples were electrophoresed with SDS-PAGE and transferred ontothe PVDF membrane (Millipore, USA) and blocked with 5 % fat-free milk, followed by overnight incubation with the primary antibodies licludinganti-GLUT1 (ab115730), anti-LDHA (ab101562), anti-HK2 (ab209847), anti-VEGF (ab32152), anti-LATS2(ab135794), anti-p-YAP (ab52771), anti-YAP (ab76252), anti-HIF-1α (ab51608) and anti-β-actin (ab8226) which were bought from Abcam (USA) and at the dilution of 1:1200. After that, the membranes were treated with an HRP-conjugated secondary antibody (Abcam, USA). The ECL kit (Solarbio, China) was applied to visualize the bands.

## 2-NBDG uptake assay

The 2-NBDG is a kind of fluorescent analog of glucose, it has been used to assess glucose transport in various cells. After the transfected A549 and H1299 cells were treated with melittin for 48h, the cells were harvested, resuspended and incubated with 100 μM 2-NBDG (Sigma, USA) for 30 min at 37. 2-NBDG levels were determined by a microscope (Olympus, Japan).

### Tubule formation assays

Matrigel (BD, USA) was added to each well of a 24-well plate and incubated until polymerization. HUVEC cells were suspended in the medium, and 0.1 mL of cell suspension was transferred to each well. The plate was incubated for 12h and the number of nodes and the completeness of the tubule per image was quantified.

### Xenograft tumor model *in vivo*

All Animal Studies followed the ARRIVE guidelines. These experiments were approved by the Animal Care and Ethics Committee. To assess tumor growth, 100 μL of A549 cells (normoxia, hypoxia, hypoxia+si-NC and hypoxia+si-LATS2) were subcutaneously injected into the flank of nude mice (4‒5-week-old, Vital River, China). Then mice were divided into 5 groups (6 mice per group). The hypoxia melittin group, hypoxia+melittin+si-NC group, and hypoxia+melittin+si-LATS2 group were administered 5 mg/kg of melittin daily. Tumor growth was monitored by caliper every 1 week; 4 weeks later, the mice were euthanized, and the tumors were collected.

### Statistical analysis

All data were exhibited as mean ± SD. The difference between two groups and multiple groups were processed by Student's *t*-test or one-way ANOVA with Bonferroni post hoc analysis respectively. All experiments were performed in triplicate; p < 0.05 reported a significant difference.

## Results

### Melittin protected against hypoxia-induced NSCLC cell proliferation

**Fig. 1:**
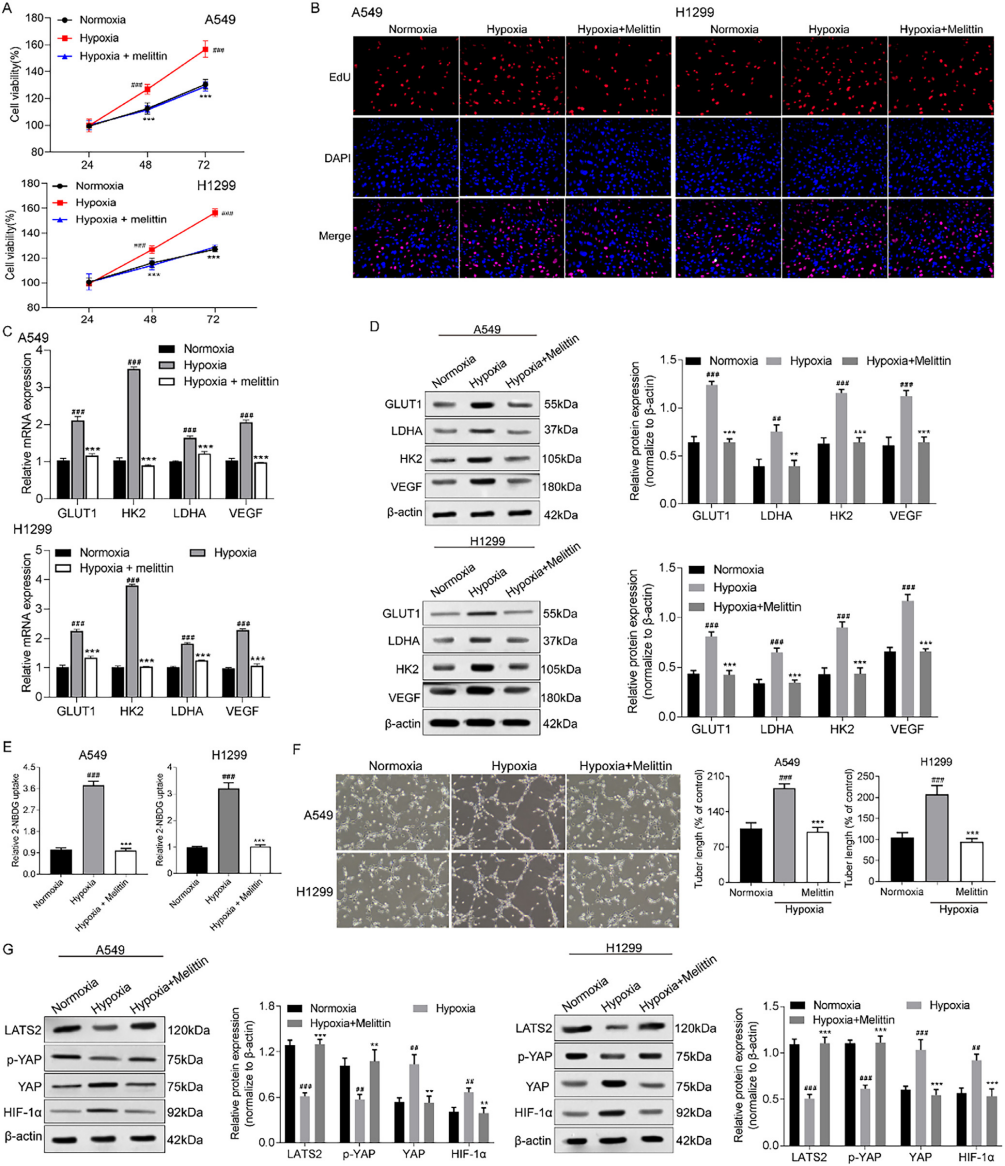
**Melittin inhibited hypoxia-induced cancer progress and the YAP/HIF-1α signaling pathway.** (A) The cell viabilities of A549 and H1299 cells were detected by MTT assay. (B) Cell proliferation was monitored by EdU assay. The mRNA and protein expression levels of GLUT1, LDHA, HK2 and VEGF were determined by (C) qRT-PCR assay and (D) western blotting assay. (E) Glycolysis was measured by the 2-NBDG uptake assay. (F) Angiogenesis was assessed by tubule formation assay. (G) The expressions of LAST2, p-YAP, YAP and HIF-1α at protein level were detected by western blotting assay. ^##^ p < 0.01, ^###^ p < 0.001 compared with normoxia group and ** p < 0.01, *** p < 0.001 compared with hypoxia group.

To examine the impact of hypoxia on cell proliferation and the protective effect of melittin against hypoxia-induced proliferation, MTT assay and EdU assay were performed. As shown in Fig. 1A, the viabilities of A549 and H1299 cells were higher after hypoxia treatment for 12 h, 24 h, 48 h than the normoxia cells, and melittin reversed the trend of cell viability under the hypoxia environment. The results of the EdU assay also showed that melittin protected against hypoxia-induced A549 and H1299 cell proliferation (Fig. 1 B).

### Melittin protected against hypoxia-induced NSCLC cell glycolysis and angiogenesis

GLUT1, LDHA and HK2 are key enzymes and catalyze many pivotal steps in glycolysis. VEGF is a crucial regulator of angiogenesis and is usually overexpressed in NSCLC.^18^^,^^19^ As shown in Fig. 1C‒D, qRT-PCR and western blot analysis showed that hypoxia treatment increased the expressions of GLUT1, LDHA, HK2 and VEGF at mRNA and protein levels compared with normoxia treated A549 and H1299 cells. Furthermore, melittin could partially abrogate hypoxia-induced up-regulation of GLUT1, LDHA, HK2 and VEGF. As expected, the results of 2-NBDG uptake and tubule formation assays strongly supported that melittin partially abrogated hypoxia-induced glycolysis and angiogenesis in NSCLC cells (Fig. 1E‒F).

### Melittin suppressed hypoxia-induced YAP binding to HIF-1α in NSCLC cells

The Hippo signaling pathway is tumor-suppressive. YAP is a transcriptional activator crucial for promoting glycolysis in hepatocellular carcinoma cells.^20^ We proposed a possibility that the Hippo signaling pathway participated in melittin protective roles against hypoxia-induced cancer progression of NSCLC. As shown in Fig. 1G, under hypoxia stimuli in A549 and H1299 cells, the protein expressions of LATS2 and p-YAP were decreased. The Hippo signaling pathway was inactivated, which promoted YAP binds to HIF-1α. After treatment with melittin, both the LATS2 and p-YAP were increased, while YAP and HIF-1α were decreased compared to hypoxia induced cells. The above results suggested that melittin regulate HIF-1α expression through the inactivation of YAP, which protected against hypoxia-induced cancer progression of NSCLC cells.

### Melittin inactivated hypoxia-induced YAP/HIF-1α signaling pathway via up-regulation of LATS2

**Fig. 2:**
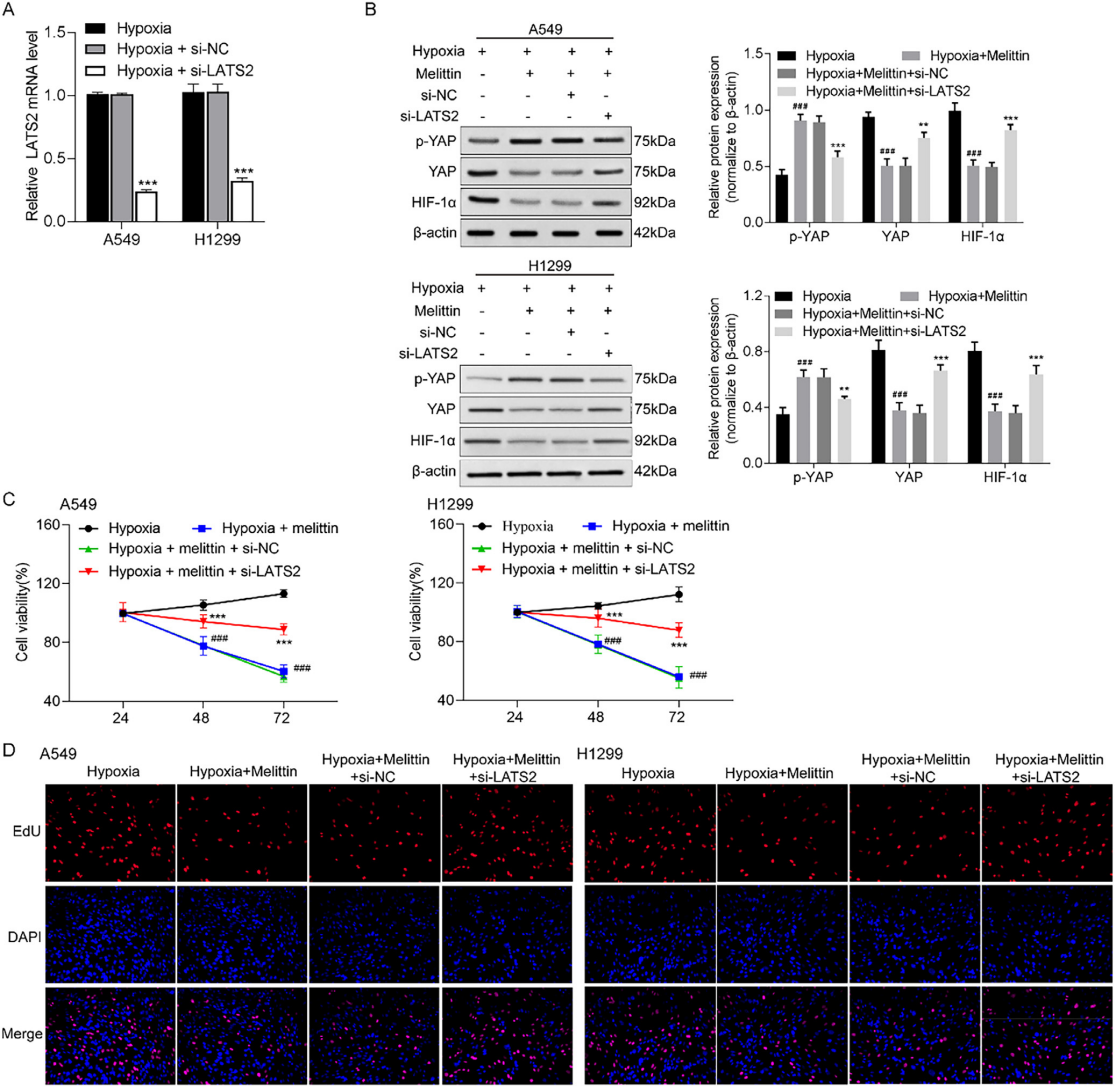
**LATS2 knockdown mediated the effects of melittin on the YAP/HIF-1α pathway, cell viability and proliferation.** (A) The LATS2 mRNA levels in A549 and H1299 cells were detected by qRT-PCR. (B) The expression levels of p-YAP, YAP and HIF-1α were analyzed by western blot assay. (C) The cell viabilities of A549 and H1299 cells were investigated by MTT assay. (D) Cell proliferation was monitored by EdU assay. ^###^ p< 0.001 compared with hypoxia group and ** p < 0.01, *** p < 0.001 compared with hypoxia+melittin+si-NC group.

To investigate the role of LATS2, the endogenous LATS2 was knocked down. The result of Fig. 2A indicated that the LATS2 expression was decreased in A549 and H1299 cells transfected with si-LATS2. Next, the cells were exposed in the hypoxia condition and divided into hypoxia, hypoxia+melittin, hypoxia+melittin+si-NC, and hypoxia+melittin+si-LATS2 groups. Western blot assay manifested that the expression of p-YAP was suppressed whereas YAP and HIF-1α were promoted with the knockdown of LATS2 compared with the hypoxia+melittin group (Fig. 2B). The above results suggested that melittin activate the expression of LATS2 to inactivate YAP/HIF-1α pathway, ultimately inhibiting cancer progression.

### LATS2 knockdown mediated the effects of melittin on hypoxia-induced cell proliferation

To confirm whether LATS2 knockdown promotes cell proliferation, we measured cell viability by MTT assay. As shown in Fig. 2C, the inhibitory cell viability of A549 and H1299 cells caused by melittin was rescued by LATS2 knockdown. The trend of cell proliferation measured by EdU assay was consistent with the above results (Fig. 2D).

### LATS2 knockdown mediated the effects of melittin on hypoxia-induced glycolysis and angiogenesis

**Fig. 3:**
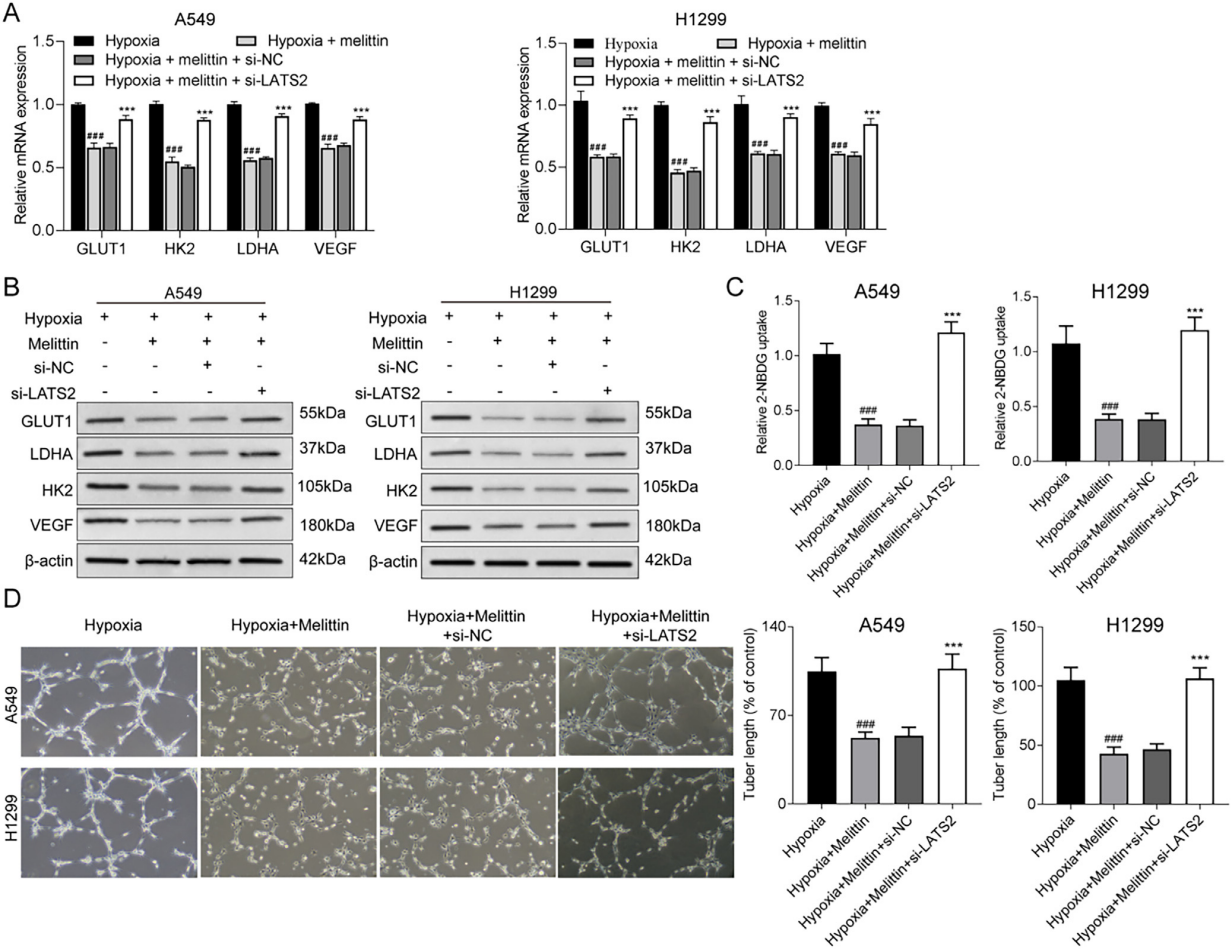
**LATS2 knockdown mediated the effects of melittin on glycolysis and angiogenesis.** The mRNA and protein expression levels of GLUT1, LDHA, HK2 and VEGF were assessed by (A) qRT-PCR assay and (B) western blot assay. (C) Glycolysis was determined by 2-NBDG uptake assay. (D) Angiogenesis was measured by tubule formation assay. ^###^ p < 0.001 compared with hypoxia group and *** p < 0.001 compared with hypoxia+melittin+si-NC group.

We further investigated glycolysis and angiogenesis. The results showed that the mRNA and protein expression levels of GLUT1, LDHA, HK2 and VEGF were augmented in the hypoxia+melittin+si-LATS2 group relative to the hypoxia+melittin group (Fig. 3A and B). The results of the 2-NBDG uptake assay and tubule formation assay also presented that LATS2 knockdown could reverse the impacts of melittin on the glycolysis and angiogenesis of NSCLC cells (Fig. 3C and D). The data of Figs. 2 and 3 testified that melittin inactivated the YAP/HIF-1α pathway via up-regulation of LATS2 to inhibit hypoxia-induced cell proliferation, glycolysis and angiogenesis in NSCLC cells.

### Melittin impeded hypoxia-induced tumor growth, glycolysis and angiogenesis *in vivo*

**Fig. 4:**
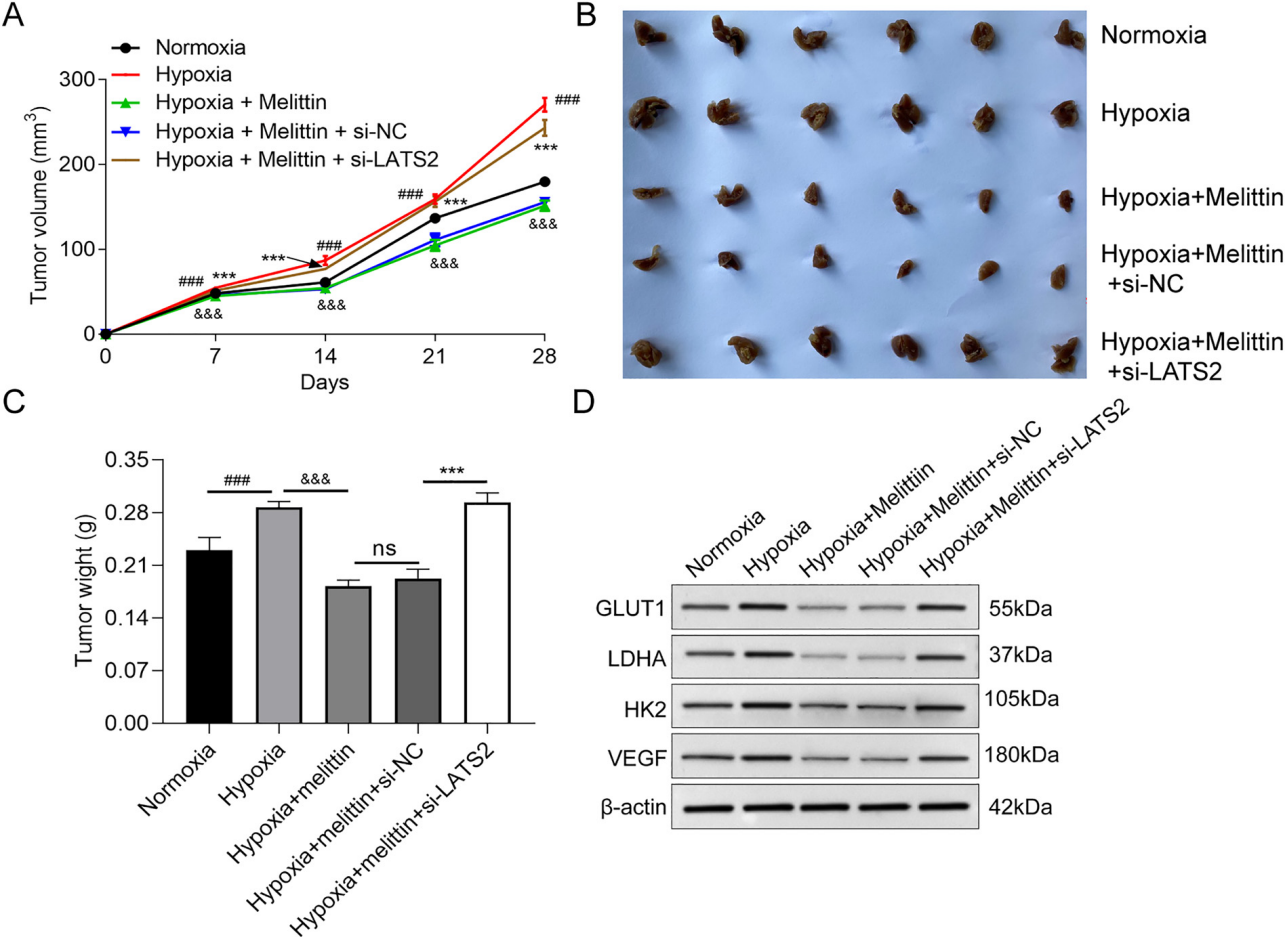
**LATS2 knockdown mediated the effects of melittin *in vivo.*** (A) Tumor volume was calculated every 7 days. (B) Photograph and (C) the average weights of the excised tumors at the endpoint. (D) The levels of GLUT1, LDHA, HK2 and VEGF in tumor tissues were detected by western blot. ^###^ p < 0.001 compared with normoxia group, ^&&&^ p < 0.001 compared with hypoxia group and *** p < 0.001 compared with hypoxia+melittin+si-NC group; *** p < 0.001; ns, no significance compared with the indicated group.

To ascertain the role of melittin *in vivo*, the following assay in nude mice was performed. The result of Fig. 4A elucidated that tumor volume in the normoxia group was notably smaller relative to that in the hypoxia group, the hypoxia+melittin group was bigger than the hypoxia group, and LATS2 knockdown promoted tumor volume. Furthermore, melittin decreased tumor growth and tumor weight when compared with the hypoxia group, LATS2 knockdown could reverse the impacts of melittin (Fig. 4B and C). Additionally, the protein levels of GLUT1, LDHA, HK2 and VEGF in tumor tissues declined with melittin treatment compared with the hypoxia group, and the levels of these proteins were remarkably increased in the hypoxia+melittin+si-LATS2 group compared to that in the hypoxia+melittin group (Fig. 4D). These data testified that melittin inhibited hypoxia-induced tumor growth, glycolysis and angiogenesis by up-regulation of LATS2 *in vivo*.

## Discussion

NSCLC is the major type of lung cancer. Hypoxia commonly exists in tumors, and cancer cells can survive and even proliferate in a hypoxic environment. Researchers reported that hypoxia in NSCLC is an important factor in survival and proliferation.^21^ These results were also confirmed in this paper. It was found in qRT-PCR, western blot, tubule formation assays and 2-NBDG uptake assay that the hypoxia environment promoted the proliferation, glycolysis and angiogenesis of NSCLC cell lines. What's more, the activation of the Hippo signaling pathway has been confirmed to be involved in the inhibitory roles of melittin on hypoxia-induced cell proliferation, glycolysis and angiogenesis.

Hippo is an evolutionarily conserved pathway in normal cells. The abnormal regulation of the Hippo signaling pathway can lead to the occurrence of tumors. As an important signaling kinase of the Hippo pathway, LATS2 enhanced the phosphorylation of YAP, which resulted in the inactivation of YAP downstream signaling by inhibiting the translocation of YAP into the nucleus.^22^^,^^23^ However, once the Hippo pathway is inactivated, YAP will transfer into the nucleus and interact with the Transcriptional Enhancer Associate Domain (TEAD). The YAP-TEAD complex will drive the expression of target genes, thus promoting cell proliferation, EMT and promoting the development of tumors.^24^^,^^25^ In addition, the hypoxia environment can inactivate the Hippo signaling pathway in hepatoma cells, promote the translocation of YAP and bind to HIF-1α in the nucleus, and maintain the stability of HIF-1α protein to accelerate glycolysis.^20^ In this study, the hypoxia-induced elevated YAP and HIF-1α were reversed by melittin. What's more, melittin suppressed hypoxia-induced A549 and H1299 cell proliferation, glycolysis and angiogenesis by blocking the YAP/HIF-1α pathway. Moreover, melittin suppressed hypoxia-induced tumor growth *in vivo*. Mammalian LATS kinases were initially identified as putative tumor suppressors in a *Drosophila* screen for regulators of organ size.^26^^,^^27^ Subsequently, studies revealed that LATS2 is a tumor suppressor gene that controls cell proliferation, cell death, and cell migration.^28^^,^^29^ In this paper, we found that melittin decreased cell proliferation, glycolysis and angiogenesis via up-regulation of LATS2, which promoted the effects of melittin on NCSLC. It was also worth noting that LATS2 knockdown completely blocked the effect of melittin *in vitro* and *in vivo*.

Taken together, melittin protects against hypoxia-induced NSCLC cell proliferation, glycolysis, and angiogenesis through inactiving YAP/HIF-1α pathway via up-regulation of LATS2. This discovery provided a novel theoretical basis for implying that melittin might become a potential natural agent against NSCLC.

## Availability of data and materials

The datasets used during the present study are available from the corresponding author upon reasonable request.

## Ethics approval

Animal experiments were approved by the Ethical Committee (Ethical NO 2020-4012) for Animal Research of the Shandong Provincial Hospital Affiliated to Shandong First Medical University and conducted based on the state guidelines of the Ministry of Science and Technology of China.

## Authors’ contributions

Hao Li did all the work and gave final approval for the version to be submitted.

## Funding

No funding was received.

## Conflicts of interest

The authors declare no conflicts of interest.

## References

[1] da Silveira MB, Lima KF, da Silva AR, Dos Santos RAS, Moraes KCM (2018). Mir-513a-3p contributes to the controlling of cellular migration processes in the A549 lung tumor cells by modulating integrin beta-8 expression. Mol Cell Biochem.

[2] Ramalingam SS, Yang JC-H, Lee CK, Kurata T, Kim D-W, John T (2018). Osimertinib As First-Line Treatment of EGFR Mutation-Positive Advanced Non-Small-Cell Lung Cancer. J Clin Oncol.

[3] Hanahan D, Weinberg RA. (2011). Hallmarks of cancer: the next generation. Cell.

[4] Weis SM, Cheresh DA. (2011). Tumor angiogenesis: molecular pathways and therapeutic targets. Nat Med.

[5] Semenza GL. (2010). HIF-1: upstream and downstream of cancer metabolism. Curr Opin Genet Dev.

[6] Zhou F, Du J, Wang J. (2017). Albendazole inhibits HIF-1alpha-dependent glycolysis and VEGF expression in non-small cell lung cancer cells. Mol Cell Biochem.

[7] Kaur B, Khwaja FW, Severson EA, Matheny SL, Brat DJ, Van Meir EG. (2005). Hypoxia and the hypoxia-inducible-factor pathway in glioma growth and angiogenesis. Neuro Oncol.

[8] Mahase S, Rattenni RN, Wesseling P, Leenders W, Baldotto C, Jain R (2017). Hypoxia-Mediated Mechanisms Associated with Antiangiogenic Treatment Resistance in Glioblastomas. Am J Pathol.

[9] Kaelin WG, Ratcliffe PJ (2008). Oxygen sensing by metazoans: the central role of the HIF hydroxylase pathway. Mol Cell.

[10] Seagroves TN, Ryan HE, Lu H, Wouters BG, Knapp M, Thibault P (2001). Transcription factor HIF-1 is a necessary mediator of the pasteur effect in mammalian cells. Mol Cell Biol.

[11] O'Donnell JL, Joyce MR, Shannon AM, Harmey J, Geraghty J, Bouchier-Hayes D. (2006). Oncological implications of hypoxia inducible factor-1alpha (HIF-1alpha) expression. Cancer Treat Rev.

[12] Tennant DA, Duran RV, Gottlieb E. (2010). Targeting metabolic transformation for cancer therapy. Nat Rev Cancer.

[13] Finley LW, Carracedo A, Lee J, Souza A, Egia A, Zhang J (2011). SIRT3 opposes reprogramming of cancer cell metabolism through HIF1alpha destabilization. Cancer Cell.

[14] Rady I, Siddiqui IA, Rady M, Mukhtar H. (2017). Melittin, a major peptide component of bee venom, and its conjugates in cancer therapy. Cancer Lett.

[15] An H-J, Kim J-Y, Kim W-H, Gwon M-G, Gu HM, Jeon MJ (2018). Therapeutic effects of bee venom and its major component, melittin, on atopic dermatitis in vivo and in vitro. Br J Pharmacol.

[16] Al-Ani I, Zimmermann S, Reichling J, Wink M (2015). Pharmacological synergism of bee venom and melittin with antibiotics and plant secondary metabolites against multi-drug resistant microbial pathogens. Phytomedicine.

[17] Gao D, Zhang J, Bai L, Li F, Dong Y, Li Q (2018). Melittin induces NSCLC apoptosis via inhibition of miR-183. Onco Targets Ther.

[18] Alevizakos M, Kaltsas S, Syrigos KN. (2013). The VEGF pathway in lung cancer. Cancer Chemother Pharmacol.

[19] Cathcart MC, Gately K, Cummins R, Drakeford C, Kay EW, O'Byrne KJ (2014). Thromboxane synthase expression and correlation with VEGF and angiogenesis in non-small cell lung cancer. Biochim Biophys Acta.

[20] Zhang X, Li Y, Ma Y, Yang L, Wang T, Meng X (2018). Yes-associated protein (YAP) binds to HIF-1alpha and sustains HIF-1alpha protein stability to promote hepatocellular carcinoma cell glycolysis under hypoxic stress. J Exp Clin Cancer Res.

[21] Costello S, Macbeth F. (1991). Management of lung cancer. BMJ.

[22] Xie J, Yu F, Li D, Zhu X, Zhang X, Lv Z. (2016). MicroRNA-218 regulates cisplatin (DPP) chemosensitivity in non-small cell lung cancer by targeting RUNX2. Tumor Biol.

[23] Yi J, Lu L, Yanger K, Wang W, Sohn BH, Stanger BZ (2016). Large tumor suppressor homologs 1 and 2 regulate mouse liver progenitor cell proliferation and maturation through antagonism of the coactivators YAP and TAZ. Hepatology.

[24] Moroishi T, Hansen CG, Guan KL. (2015). The emerging roles of YAP and TAZ in cancer. Nat Rev Cancer.

[25] Zhao B, Wei X, Li W, Udan RS, Yang Q, Kim J (2007). Inactivation of YAP oncoprotein by the Hippo pathway is involved in cell contact inhibition and tissue growth control. Genes Dev.

[26] Justice RW, Zilian O, Woods DF, Noll M, Bryant PJ. (1995). The Drosophila tumor suppressor gene warts encodes a homolog of human myotonic dystrophy kinase and is required for the control of cell shape and proliferation. Genes Dev.

[27] Xu T, Wang W, Zhang S, Stewart RA, Yu W. (1995). Identifying tumor suppressors in genetic mosaics: the Drosophila lats gene encodes a putative protein kinase. Development.

[28] Murakami H, Mizuno T, Taniguchi T, Fujii M, Ishiguro F, Fukui T (2011). LATS2 is a tumor suppressor gene of malignant mesothelioma. Cancer Res.

[29] Furth N, Bossel Ben-Moshe N, Pozniak Y, Porat Z, Geiger T, Domany E (2015). Down-regulation of LATS kinases alters p53 to promote cell migration. Genes Dev.

